# Supplementary material for: Causal Relationship between Aging and Anorexia Nervosa: A White-Matter-Microstructure-Mediated Mendelian Randomization Analysis
Source: Biomedicines. 2024 Aug 16;12(8):1874. doi: 10.3390/biomedicines12081874 (PMC11351342; doi:10.3390/biomedicines12081874)
Supplement: Supplementary file 1 [file biomedicines-12-01874-s001.zip › Supplementary Figure/Figure S3/Funnel plot of MR analysis for TL to ubm-b-1955.pdf]

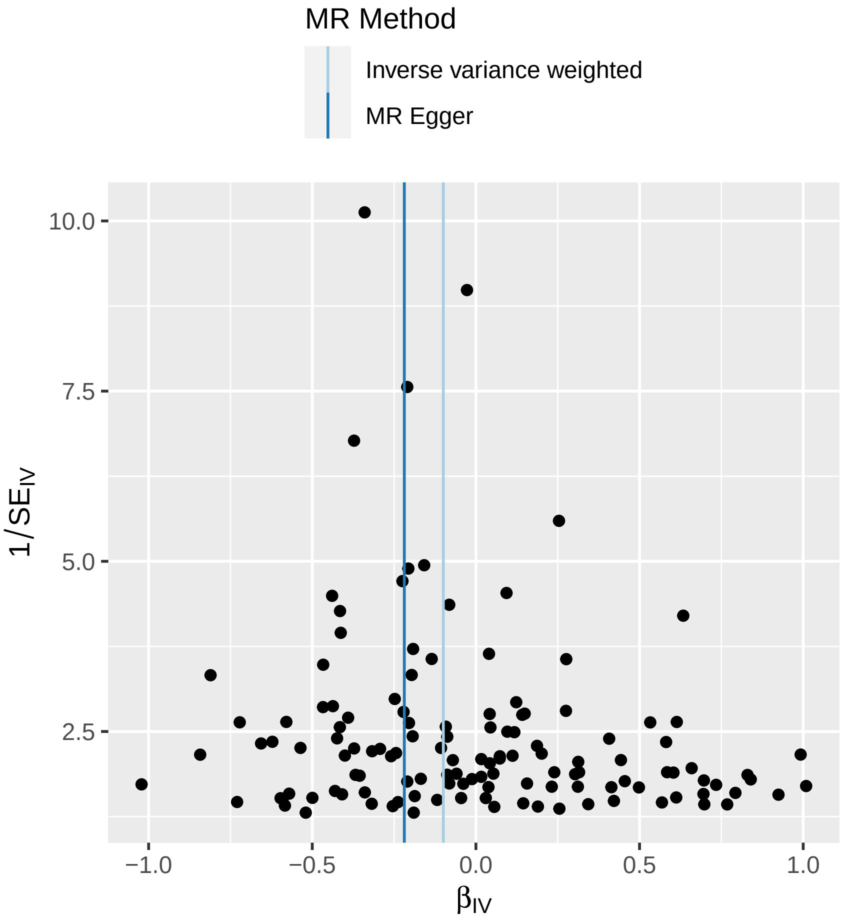
MR Method
Inverse variance weighted
MR Egger
10.0
7.5
5.0
2.5
1/SE$_{IV}$
−1.0
−0.5
0.0
0.5
1.0
β_{IV}
